# First report of spondylodiscitis caused by *Bacillus circulans* in an immunocompetent patient: Clinical case and review of the literature

**DOI:** 10.1016/j.idcr.2021.e01058

**Published:** 2021-01-26

**Authors:** Alessandro Russo, Umberto Tarantino, Gabriella d’Ettorre, Carlo Della Rocca, Giancarlo Ceccarelli, Elena Gasbarra, Mario Venditti, Riccardo Iundusi

**Affiliations:** a Policlinico “Umberto I”, Department of Public Health and Infectious Diseases, “Sapienza” University of Rome, Italy; b Department of Orthopaedics and Traumatology, University of Rome “Tor Vergata”, "Policlinico Tor Vergata" Foundation, Rome, Italy; c Department of Medico-Surgical Sciences and Biotechnology, Polo Pontino, “Sapienza” University of Rome, Italy

**Keywords:** Bacillus circulans, Spondylodiscitis, Biopsy, First case

## Abstract

*Bacillus circulans* is mainly considered an opportunistic pathogen in immunocompromised patients. However, many different infections have been described in the literature: bacteremia, abscesses, meningitis, endophthalmitis, and wound infections.

We observed a spondylodiscitis caused by *Bacillus circulans* in an immunocompetent patient. To date, this is the first case reported in literature. Vertebral osteomyelitis represents for clinicians a challenging infection to manage and treat, because of its insidious and indolent course. The diagnosis is frequently difficult and can often be delayed for several months and initially be misdiagnosed and mismanaged. For this reason, the clinical case was described and all published cases of infection caused by *Bacillus circulans* were reviewed.

## Highlights

• Vertebral osteomyelitis represents for clinicians a challenging infection to manage and treat.
• *Bacillus circulans* is mainly considered as a rare, opportunistic pathogen in immunocompromised patients.
• We report a spondylodiscitis caused by *Bacillus circulans* in an immunocompetent patient.
• To date, this is the first case reported in literature.
• The clinical case was described and all published *Bacillus circulans* infections were reviewed

## Introduction

*Bacillus circulans* is a gram-positive, rod-shaped organism. It is motile via peritrichous flagella and produces endospores. *Bacillus circulans* is found in soil, sewage, food, and infant bile. This bacterium is also isolated from gut of bee larvae [1].

In the literature, few cases of infection caused by *Bacillus circulans* are described. It is mainly considered as an opportunistic pathogen in immunocompromised patients. However, many different infections have been reported: bacteremia, abscesses, meningitis, endophthalmitis, and wound infections.

Recently, we observed a spondylodiscitis caused by *Bacillus circulans* in an immunocompetent patient. To date, this is the first case reported in literature. For this reason, we described the clinical case and reviewed all published cases of infection caused by *Bacillus circulans*.

## Case report

A 65-year-old Caucasian man with a history of hypertension, was evaluated in February 2020 for prolonged lower back pain. Before the evaluation, the patient reported a history of low-grade fever for 1 month associated with severe diarrhea for 2 weeks in the previous 2 months. At the visit, blood exams showed a C-reactive protein (CRP) of 15 mg/l (normal value <5.0). He underwent a lumbar spine magnetic resonance imaging (MRI) that highlighted a suspected spondylodiscitis at L5-S1. Empirical antibiotic therapy with amoxicillin/clavulanate plus minocycline was started after percutaneous bone biopsy, without any improvement of symptoms and reduction of CRP value. After biopsy, blood cultures were collected. All other possible etiologies of spondylodiscitis were excluded (i.e., *Mycobacterium tuberculosis*, *Brucella* spp). A transthoracic echocardiography was performed, excluding the presence of endocarditis. Bone culture resulted sterile and patient was treated for 6 weeks with the same antibiotic regimen.

**Fig. 1:**
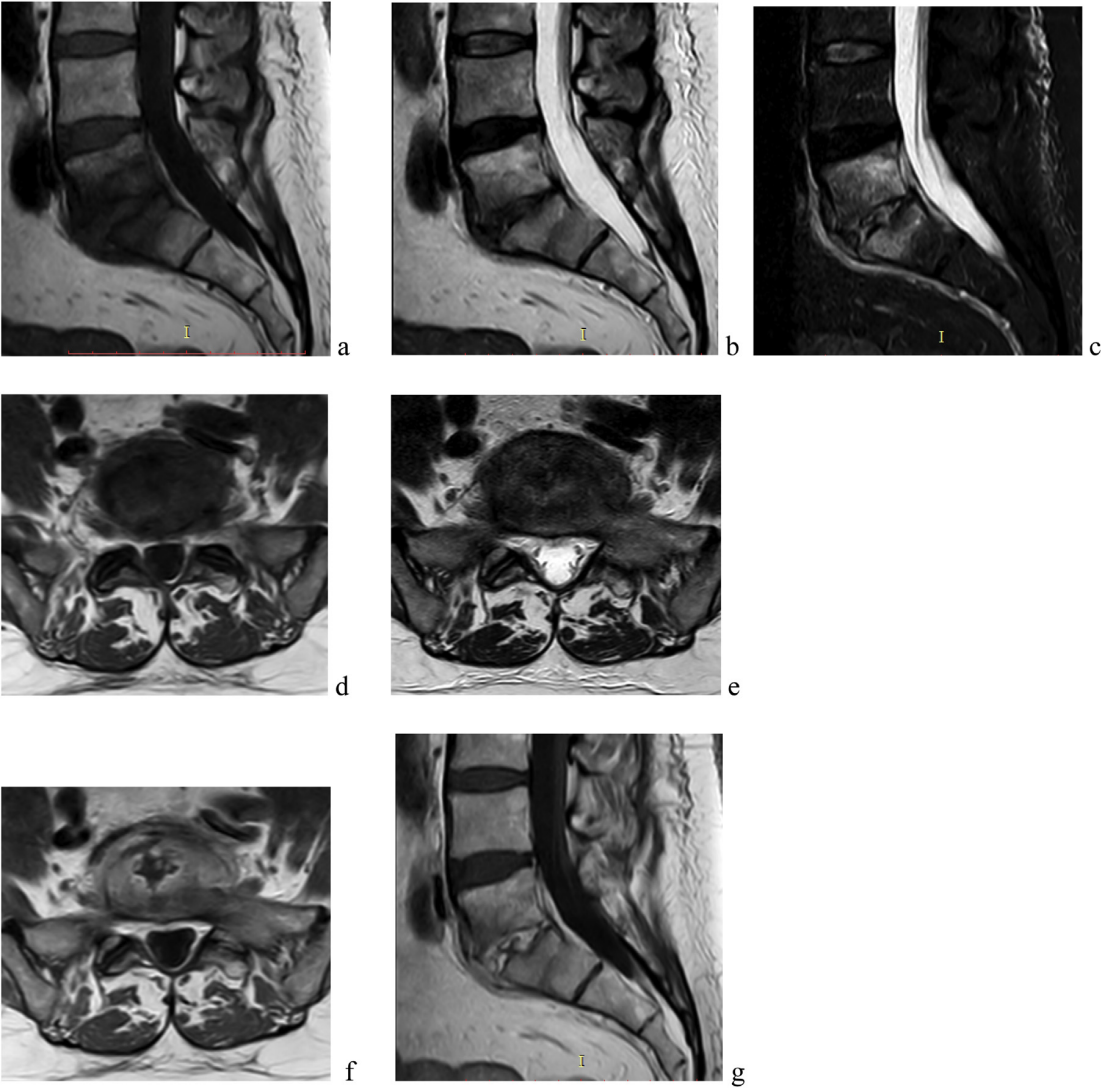
Lumbar spine MRI at time of *Bacillus circulans* isolation.

On the third month, clinical symptoms did not improve. Worsening lower back pain and a repeat increment of CRP required a new infectious diseases consultation. A new lumbar spine MRI was performed, showing the persistence of heterogeneous lesions in L5 and S1 with increased signal in T2-weighted (Fig. 1**-b,e**), STIR-weighted images (Fig. 1**-c**), hypo-signal in T1-weighted images (Fig. 1**-a,d**). The changes were registered at L5 and S1 vertebral bodies as bone edema and L5-S1 intervertebral disc as increased water content. After contrast administration, the images were highly suggestive for spondylodiscitis L5-S1 (Fig. 1**-f,g**). No findings of inflammatory purulent process of the iliopsoas muscles and/or paraspinal or subdural/epidural phlegmon or abscess were detected.

**Fig. 2:**
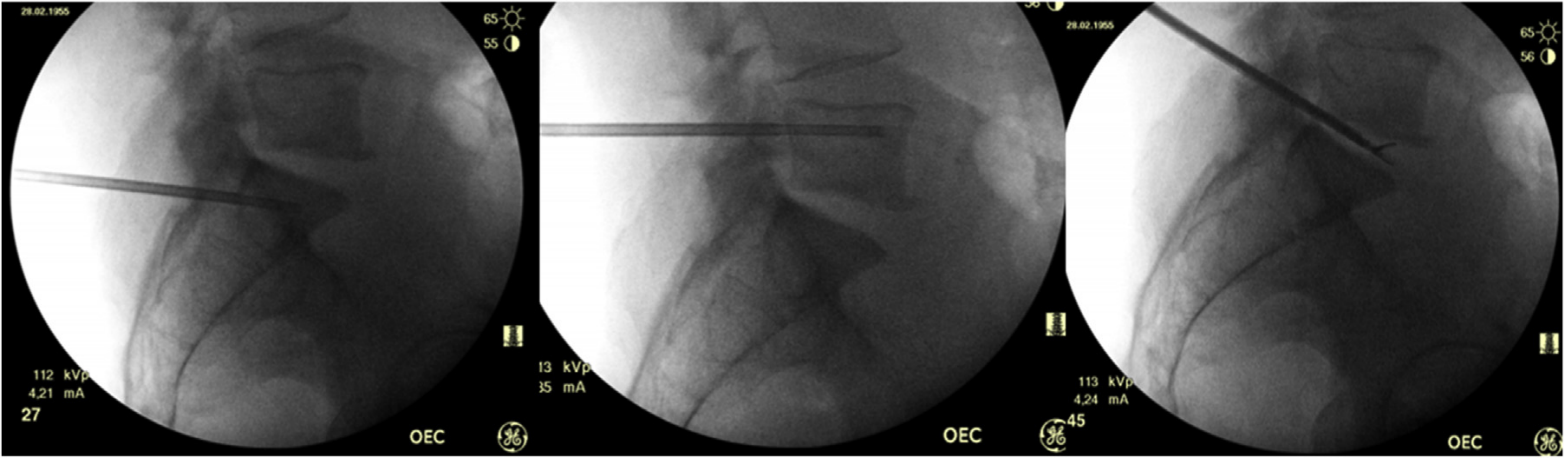
Intra-operative fluoroscopy of bone and disc biopsy.

Considering the clinical, radiological and laboratoristic findings, it was decided to perform another vertebral and disc biopsy. The patient was positioned in the prone position on the radiological table with the spine extended to increase the disc space in L5-S1. Two small skin incisions were made: one access for both L5 and S1 pedicles and the other, more lateral, for L5-S1 trans-foraminal disc approach. Unilateral transpedicular approach was performed with a 13-gauge bone biopsy needle under fluoroscopical guidance (OEC Brivo Plus, GE Healthcare, USA). The needle was introduced first into L5 body through the left pedicle and bone biopsy was performed; another sterile needle was introduced into S1 body always through the left pedicle and a bone biopsy was obtained. At last, transforaminal L5-S1 disc biopsy was performed using percutaneous endoscopic forceps through an 11-gauge working cannula on the left side (Fig. 2). No intra- or post-procedure complications occurred. Left approaches were chosen because of previous L5 at the right side transpeduncolar biopsy 3 months before, that resulted negative. The duration of the whole procedure was about 70 min. Patient was discharged the day after.

Cultures of bone and disc showed the growth of *Bacillus circulans*. The organism was identified using MALDI-TOF mass spectrometry (MS) (Bruker Daltonics) and the Vitek 2 automated system (bioMérieux, Marcy l’Etoile, France) for antimicrobial susceptibility testing. Minimum inhibitory concentrations (MICs) were established according to the European Committee on Antimicrobial Susceptibility Testing (EUCAST) breakpoints. The strain showed susceptibility to erythromycin, vancomycin, chloramphenicol, gentamicin and levofloxacin. It showed resistance to penicillin, clindamycin, tetracycline, cefazolin and cefotaxime.

After 2-week treatment with intravenous teicoplanin, the patient completed 4 weeks of oral levofloxacin, 750 mg once daily. At the end of therapy, the CRP value normalized with a complete resolution of clinical symptoms. A ^18^F-Fluorodeoxyglucose positron emission tomography/computed tomography (FDG-PET/CT), after 1 month, resulted negative.

## Discussion and review of literature

To our knowledge, this is the first case described in the literature of vertebral osteomyelitis (VO) caused by *Bacillus circulans*.

*Bacillus circulans* is considered as an opportunistic pathogen [1]; in the described cases it was also recognized as the etiology of endocarditis, abscesses, endophthalmitis, and meningitis in humans as reported in Table 1. Moreover, a case of sepsis of unknown origin in an immunocompromised patient was described in 2011, which later led to patients’ death. Finally, it was also associated with wound infection in 1985. In the literature there are reported 12 cases of documented infection caused by *Bacillus circulans* [[2], [3], [4], [5], [6], [7], [8], [9], [10], [11], [12], [13]], and 3 cases in which *Bacillus circulans* was considered a possible etiology of infection [14].

**Table 1:** Patients with *Bacillus circulans* infections reported in the literature.

First Author, year	Age, sex	Underlying conditions	Type of infection	Complications	Therapy	Outcome
Boyette, 1952	5 days, male	–	Meningitis	Septic shock	Penicillin	Death
Logan, 1985	78 years, female	Ovarian carcinoma	Surgical wound infection	–	Cotrimoxazole	Cure
Roncoroni, 1985	N/A	N/A	Cerebrospinal fluid ventriculo-atrial shunt infection	*Bacillus larvae* infection	Cefotaxime, cotrimoxazole and rifampicin	Improvement
Banerjee, 1988	N/A	N/A (acute leukemia and metastatic breast cancer)	Suspected catheter-related bacteremia	N/A	N/A	N/A
Banerjee, 1988	N/A	N/A (acute leukemia and metastatic breast cancer)	Suspected catheter-related bacteremia	N/A	N/A	N/A
Banerjee, 1988	N/A	N/A (acute leukemia and metastatic breast cancer)	Suspected catheter-related bacteremia	N/A	N/A	N/A
Wilde, 1988	N/A	N/A	Early prosthetic knee infection (1 month)	–	Tobramycin/Gentamicin (3 weeks)	Cure
Gatermann, 1991	58 years, male	Alcohol abuse	Native aortic valve endocarditis	Heart failure, aortic valve replacement	Penicillin G + tobramycin	Cure
Goudswaard, 1995	48 years, male	Human bite wound	Proximal interphalangeal joint	–	Clindamycin	Cure
Castagnola, 1997	Unknow, children	Neuroblastoma	Catheter-related bacteraemia	–	–	Cure
Krause, 1999	56 years, Female	Prosthetic valve	Late endocarditis (15 months)	–	Teicoplanin + gentamicin (1 week) followed by trimethoprim + ciprofloxacin (6 weeks)	Cure
Fraco, 2001	80 years, female	Cataract surgery	Endophthalmitis	Recurrent uveitis	Intravitreal vancomycin and gentamicin	Improvement
Berry, 2004	62 years, male	Continuous ambulatory peritoneal dialysis, diabetes	Peritonitis	–	Intraperitoneal vancomycin + and gentamicin	Cure
Alebouyeh, 2011	62 years, male	End- stage renal disease	Bacteremia	Septic shock	Metronidazole and piperacillin- tazobactam	Death
Sanyal, 2015	60 years, male	Malnutrition	Cellulitis	–	Imipenem	Cure
Russo, 2020	65 years, male	Hypertension	Lumbar vertebral column	–	Moxifloxacin	Cure

**Legend.** N/A: not available.

Of importance, *Bacillus* spp are rarely implicated in infections and are frequently isolated as a culture contaminant. However, spores are found ubiquitously including in the hospital environment. The production of toxins and tissue invasion contribute to a range of diseases. Of interest, the spectrum of infections and susceptibility to antibiotics result similar to *Bacillus cereus* [1]. For this reason, a careful clinical evaluation is crucial to assess the significance of the isolation of a *Bacillus* organism from cultures. Clinical infections caused by *Bacillus* spp can mainly be categorized into gastrointestinal and non-gastrointestinal diseases; the latter are usually the etiology of local or systemic infections [1]. Although *Bacillus* spp. have not been recognized as major human pathogens, with recent advances in medical technology and an increased number of immunosuppressed patients they have been increasingly recognized as opportunistic pathogens especially in the hospitalized patient. Our patient reported a severe diarrhea for 2 weeks in the previous 2 months before diagnosis of spondylodiscitis. Probably, gastrointestinal inflammation with translocation of *Bacillus circulans* was favored to be the source of the lumbar osteomyelitis.

In the literature, in immunocompetent patients were described few cases of acute and chronic osteomyelitis caused by *Bacillus cereus*, the most important pathogen for humans among *Bacillus* spp. Interestingly, Fritzell and coworkers reported an experience about the detection of bacterial DNA in painful degenerated spinal discs. This in turn may be in patients without signs of clinical infection. One patient with disc hernia harbored DNA homologous to *Bacillus cereus*, and the authors concluded that 16S rRNA PCR can be a useful tool in search of bacterial DNA in degenerated discs, which in turn may be indicative of low-grade infection, manifesting itself only as pain rather than as clinical infection [15].

In conclusion, this clinical case represents a step toward a better knowledge of *Bacillus* spp, including *Bacillus circulans* and its related infections. As reported in the literature, *Bacillus* spp may be associated with indolent and/or chronic infections, also involving bone and causing osteomyelitis. Most cases of *Bacillus circulans* infection are associated with hospitalization in immunodeficient patients as reported in Table 1; however, we reported here a first case observed in immunocompetent patients from the community using a high-standard quality methodology for bone and disc biopsy.

## Author statement

On behalf of all authors, I declare no conflict of interest for the manuscript “First report of spondylodiscitis caused by *Bacillus circulans* in an immunocompetent patient: clinical case and review of the literature”.

## Funding

No funding.

## Concent

Consent: Written informed consent was obtained from the patient for publication of this case report and accompanying images. A copy of the written consent is available for review by the Editor-in- Chief of this journal on request

## Ethical approval

None.

## Author contribution

Please specify the contribution of each author to the paper, e.g. study design, data collections, data analysis, writing, others, who have contributed in other ways should be listed as contributors. Alessandro Russo and Riccardo Iundusi collected data and wrote the paper; Umberto Tarantino, Gabriella d’Ettorre, Carlo Della Rocca, Giancarlo Ceccarelli, Elena Gasbarra, Mario Venditti wrote and revised the paper.

## Declaration of Competing Interest

The authors report no declarations of interest.

## References

[1] Drobniewski F.A. (1993). Bacillus cereus and related species. Clin Microbiol Rev.

[2] Boyett D.P., Rights F.L. (1952). Heretofore undescribed aerobic spore forming bacillus in child with meningitis. J Am Med Assoc.

[3] Castagnola E., Conte M., Venzano P., Garaventa A., Viscoli C., Barretta M.A. (1997). Broviac catheter-related bacteraemias due to unusual pathogens in children with cancer: case reports with literature review. J Infect.

[4] Krause A., Gould F.K., Forty J. (1999). Prosthetic heart valve endocarditis caused by Bacillus circulans. J Infect.

[5] Berry N., Hassan I., Majumdar S., Vardhan A., McEwen A., Gokal R. (2004). Bacillus circulans peritonitis in a patient treated with CAPD. Perit Dial Int.

[6] Sanyal S.K., Karmaker M., Sultana M., Hossain M.A. (2015). Association of Bacillus circulans with non-diabetic foot infection in Bangladeshi patient. Indian J Med Microbiol.

[7] Gatermann S., Mitusch R., Djonlagic H., Hollandt H., Marre R. (1991). Endocarditis caused by Bacillus circulans. Infection.

[8] Goudswaard W.B., Dammer M.H., Hol C. (1995). Bacillus circulans infection of a proximal interphalangeal joint after a clenched-fist injury caused by human teeth. Eur J Clin Microbiol Infect Dis.

[9] Alebouyeh M., Gooran Orimi P., Azimi-Rad M. (2011). Fatal sepsis by Bacillus circulans in an immunocompromised patient. Iran J Microbiol.

[10] Tandon A., Tay-Kearney M.L., Metcalf C., McAllister L. (2001). Bacillus circulans endophthalmitis. Graefes Arch Clin Exp Ophthalmol.

[11] Logan N.A., Old D.C., Dick H.M. (1985). Isolation of Bacillus circulans from a wound infection. J Clin Pathol.

[12] Wilde A.H., Ruth J.T. (1988). Two-stage reimplantation in infected total knee arthroplasty. Clin Orthop Relat Res.

[13] Roncoroni A., Rivas M., Smayevsky J., Bianchini H., Zucarro G. (1985). Infection of a cerebrospinal fluid shunt system by Bacillus circulans and Bacillus larvae. Rev Argent Microbiol.

[14] Banerjee C., Bustamante C.I., Wharton R., Talley E., Wade J.C. (1988). Bacillus infections in patients with cancer. Arch Intern Med.

[15] Fritzell P., Bergström T., Welinder-Olsson C. (2004). Detection of bacterial DNA in painful degenerated spinal discs in patients without signs of clinical infection. Eur Spine J.

